# Comparison of the effects of ketamine via nebulization versus different pharmacological approaches in pediatric sedation: a systematic review and meta-analysis of randomized controlled trials

**DOI:** 10.1186/s12871-023-02298-4

**Published:** 2023-11-16

**Authors:** Xiao Liu, Bingchen Lang, Linan Zeng, Liang Huang, Shouming Chen, Zhi-Jun Jia, Guo Cheng, Qin Yu, Lingli Zhang

**Affiliations:** 1 Department of Pharmacy, The Third People’s Hospital of Chengdu, The Affiliated Hospital of Southwest Jiaotong University, Chengdu, China; 2 Department of Pharmacy, West China Second University Hospital, Sichuan University, Chengdu, China; 3 Evidence-Based Pharmacy Center, West China Second University Hospital, Sichuan University, Chengdu, 610041 China; 4 National Medical Products Administration (NMPA) Key Laboratory for Technical Research on Drug Products In Vitro and In Vivo Correlation, Chengdu, China; 5 Key Laboratory of Birth Defects and Related Diseases of Women and Children, Ministry of Education, Chengdu, China; 6 Department of Anesthesiology, West China Second University Hospital, Sichuan University, Chengdu, China; 7 West China School of Pharmacy, Sichuan University, Chengdu, China; 8 Department of Pediatrics, West China Second University Hospital, Sichuan University, Chengdu, China; 9 Laboratory of Molecular Translational Medicine, Center for Translational Medicine, Sichuan University, Chengdu, China; 10 National Drug Clinical Trial Institute, West China Second University Hospital, Sichuan University, Chengdu, China; 11 Chinese Evidence-based Medicine Center, West China Hospital, Sichuan University, Chengdu, China

**Keywords:** Nebulization, Ketamine, Sedation, Pediatrics, Meta-analysis

## Abstract

**Background:**

Nebulized drug delivery is commonly used in pediatric clinical practice. The growing number of literatures have reported the application of nebulized ketamine in pediatric sedation in recent years. This meta-analysis of randomized controlled trials comparing the efficacy and safety of nebulized ketamine versus different pharmacological approaches was conducted to estimate the effects of this technique in pediatric sedation.

**Methods:**

We searched PubMed, Embase, and Cochrane Library from inception to Feb 2023. All randomized controlled trials used nebulized ketamine as presurgical and pre-procedural sedatives in children were included. Sedative effects and various adverse events were considered as the outcomes.

**Results:**

Ten studies with 727 pediatric patients were enrolled. Compared to nebulized dexmedetomidine, using of ketamine via nebulization showed similar sedation satisfaction (54.79% vs. 60.69%, RR = 0.88, with 95%CI [0.61, 1.27]), success rate of parental separation (57.27% vs. 73.64%, RR = 0.81, with 95%CI [0.61, 1.08]), and mask acceptability (37.27% vs. 52.73%, RR = 0.71, with 95%CI [0.45, 1.10]). However, the using of combination of two medications (nebulized ketamine plus nebulized dexmedetomidine) was associated with better sedative satisfaction (33.82% vs. 68.11%, RR = 0.50, with 95%CI [0.27, 0.92]) and more satisfactory mask acceptance (45.59% vs. 71.01%, RR = 0.69, with 95%CI [0.56, 0.86]). Compared with nebulized ketamine, using of nebulized dexmedetomidine was associated with less incidence of emergence agitation (18.18% vs. 3.33%, RR = 4.98, with 95%CI [1.88, 13.16]).

**Conclusions:**

Based on current evidences, compared to nebulized dexmedetomidine, nebulized ketamine provides inconspicuous advantages in pediatric sedation, and it has a relatively high incidence of emergence agitation. Combination of nebulized ketamine and dexmedetomidine might be considered as one preferred option in pediatric sedation as it can provide more satisfactory sedative effects. However, there is insufficient evidence regarding nebulized ketamine versus ketamine administered through other routes and nebulized ketamine versus other sedatives. The overall low or moderate quality of evidence evaluated by the GRADE system also calls for more high-quality studies with larger sample sizes in future.

**Research registration:**

The protocol of present study was registered with PROSPERO (CRD42023403226).

**Supplementary Information:**

The online version contains supplementary material available at 10.1186/s12871-023-02298-4.

## Introduction

Relieving preoperative anxiety in pediatric patients remains an ongoing challenge for pediatric clinicians [1], and procedural sedation/analgesia (PSA) regimens always involve intravenous administration of sedatives. However, peripheral intravenous (IV) insertion is frequently cited as a primary cause of pain in children and is consistently linked to anxiety and distress [2]. In light of the increasing demand for PSA in children before various procedures or surgeries, exploring a pain-free alternative to IV insertion in pediatric sedation should be served as an important goal for clinicians.

Nebulization therapy is a popular approach to treating pediatric patients [3]. It carries a lower risk of adverse events compared to other routes of administration (such as intramuscular injection, intravenous injection, etc.) [4, 5]. In addition, ease of administration, superior patient compliance, and the relatively small drug volume required for effect make it a highly recommended option [6]. A series of aerosolized medications, including corticosteroids, ketamine, magnesium, lidocaine, and non-steroidal anti-inflammatory drugs (NSAIDs), have proven effective in various treatments [7–9].

As a traditional non-competitive N-Methyl-D-Aspartate antagonist (NMDA), ketamine has been commonly applied as presurgical and pre-procedural sedatives in children [10, 11]. It provides analgesic properties owing to its ability to antagonize NMDA receptors, reduces the levels of proinflammatory mediators during acute phase, and affects other non-NMDA pathways which are instrumental in pain and mood regulation [12].

In recent years, there have been a series of reports on use of ketamine nebulization as a preoperative sedation for pediatric patients [13, 14]. Given that, we conduct a meta-analysis from the published randomized controlled trials comparing the efficacy and safety of nebulized ketamine versus different pharmacological approaches to evaluate the effects of this technique in pediatric sedation and to provide a comprehensive understanding about its benefits and drawbacks.

## Methods

### Protocol and registration

The present meta-analysis was conducted in accordance with the Preferred Reporting Items for Systematic Reviews and Meta-Analyses (PRISMA) statement [15] and Cochrane Handbook guidelines. And we registered the protocol for this review on the International Prospective Register for Systematic Reviews (PROSPERO) (https://www.crd.york.ac.uk/prospero, CRD42023403226).

### Search strategy

Two authors (BL and SC) conducted a systematic search from electronic databases including PubMed, Embase, and Cochrane Library, covering the period from inception up to Feb 27, 2023. In addition, academic search engine Google Scholar was utilized as the additional information source. “Infant”, “child”, “adolescent”, “aerosoli*”, “nebuli*”, “ketamine” and “randomized controlled trial” were considered as the search terms (Appendix S1). The human studies without language limitation were considered in our present study.

### Eligibility criteria

#### Participants

The participants of present study were children (< 18 years old) who underwent various presurgical and pre-procedural sedation.

#### Intervention

Using ketamine via nebulization (e.g., administered with a nasal mucosal atomizer device, nebulizer, or spray) as premedication were considered as intervention.

#### Comparisons

Using ketamine via other route or using different pharmacological approaches as premedication were considered as comparisons.

#### Outcome measures

Consensus exists regarding the optimal characteristics of pediatric sedation, including successful separation from parents, achievement of anesthesia induction or facemask compliance, rapid onset and recovery, and minimal adverse effects. Therefore, we identified (1) number of patients who achieved a satisfactory level of sedation sufficient for procedures (venipuncture, diagnostics, surgical procedures, etc.)., (2) the number of children with satisfactory separation from parents and (3) the number of children with satisfactory mask acceptance as the co-primary outcomes. Onset of sedation, recovery time, and the incidence of adverse events (e.g., vomiting, nystagmus, abnormal movement, hypersalivation, hypotension, bradycardia, sneezing, coughing and emergence agitation) were considered as the secondary outcomes.

#### Study design

Only randomized controlled trials (RCTs) were considered in our present study.

#### Exclusion criteria

Reviews, conference abstracts, letters, cases, comments, preclinical studies, protocol, ongoing trials, studies performed in adults and studies with inappropriate comparisons or unrelated outcomes were excluded by us.

### Data extraction, and assessment of the risk of bias

Two authors (BL and SC) conducted literature screening and data extraction independently, followed by crosschecking with each other. Duplicated items from different databases were removed, and irrelevant records were excluded after scrutinizing their titles and abstracts. Then we perused the original texts of remaining records when information could not be ascertained. We collected the general characteristics of all studies that met the criteria (Table 1). The Cochrane risk of bias tool [16] was used to evaluate the risk of bias in RCTs based on the following aspects: random sequence generation (generation of the randomization sequence), allocation concealment, blinding of outcome assessment, incomplete outcome data, and selective reporting. Clinical research was categorized as having low, high, or unclear risk of bias based on these domains mentioned above. In the case of any disagreement, a third investigator was consulted to resolve the issue.

### Grading the quality of evidence

The Grading of Recommendations Assessment, Development, and Evaluation (GRADE) methodology [17] was employed to appraise the quality of evidence and potency of recommendations, taking into account the risk of bias, inconsistency, indirectness, imprecision, and publication bias. The quality of evidence was classified as high, moderate, low, or very low, and the analysis was conducted by using the GRADE profiler software (version 3.6, provided by the Cochrane collaboration) [18].

### Statistical analysis

The statistical analysis was conducted by using Review Manager software (Version 5.3.3, the Cochrane Collaboration 2014, the Nordic Cochrane Centre). Continuous variables were estimated by using the mean difference (MD) with a 95% confidence interval (CI). The risk ratio (RR) with a 95% confidence interval (CI) and the Mantel-Haenszel method were used to analyze dichotomous data. Heterogeneity was assessed by *I*-squared (*I*^2^) test [19]. In cases where significant heterogeneity was detected (present at *I*^2^ > 50%), the random effects model was applied, and a sensitivity analysis was conducted by omitting each study separately; otherwise, the fixed-effects model would be considered. Begg’s test and Egger’s test were used to evaluate publication bias if the number of included studies over ten [20]. The tests were conducted using version 1.2.4 of the metabias program and Stata/MP 12.0 for Windows (StataCorp LP, 4905 Lakeway Drive, College Station, TX 77,845, USA). A less-than 0.05 *P* value was considered statistically significant.

## Results

### Literature search results

**Fig. 1:**
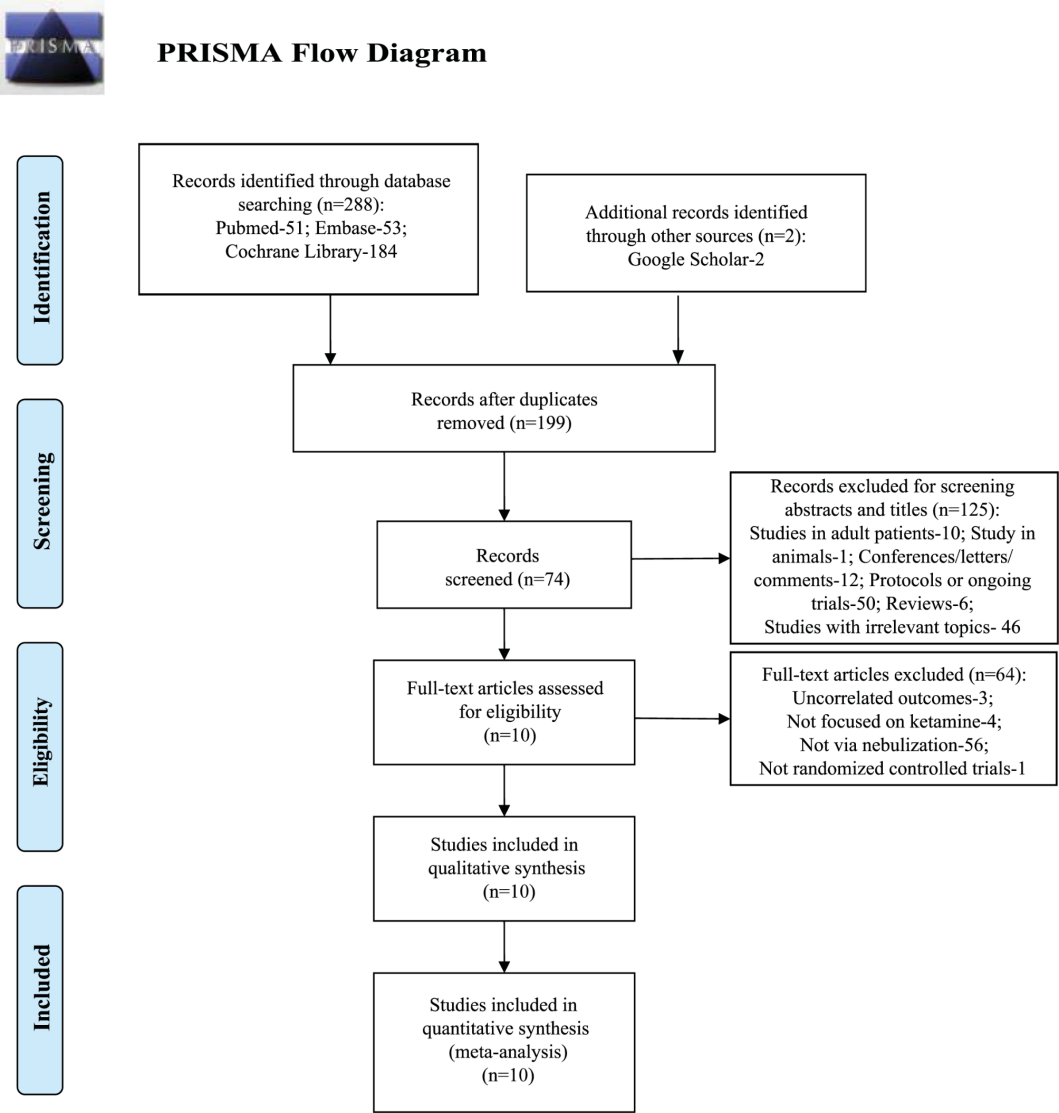
PRISMA flow diagram showing literature search results

A total of 290 studies were identified initially after screening various databases and searching additional sources. Subsequently, 199 duplicate records were removed, and 125 records were excluded by a thorough review of titles and abstracts. In these 125 excluded items, 10 were focused on adult patients, 1 was conducted on animals, 12 were conference abstracts, comments notes or letters, 50 were protocols or ongoing trials, 6 were reviews, and 46 were studies with irrelevant topics. Consequently, 64 items were further excluded following full-text review, and 3 of them were not relevant to the outcomes of the study, 4 of them did not focus on ketamine, 56 of them focused on ketamine administered not via nebulization, and 1 of them was not a randomized controlled trial. Eventually, 10 studies were selected for subsequent analysis [14, 21–29]. The PRISMA flowchart (Fig. 1) provides details on the identification of the literature.

### Basic characteristics of enrolled studies

The involved studies were published from 2015 to 2022, with a total of 727 eligible pediatric patients ranging in age from 1 to 12 years. Among the included studies, six examined the effects of nebulized ketamine versus nebulized dexmedetomidine, while three focused on the effects of nebulized ketamine versus nebulized midazolam. Furthermore, three studies examined the effects of nebulized ketamine versus combination of nebulized ketamine and dexmedetomidine. In addition, one study reported findings on effects of nebulized ketamine versus oral ketamine, and one study examined the effects of nebulized ketamine versus intravenous ketamine. An overview of the main characteristics of the enrolled studies was presented in Table 1 including the following information: first author, publication year, range of age, American Society of Anesthesiologists status, type of surgery/procedure, drug dosage, sample size, scale used for sedation measurement and outcomes.

**Table 1:** The general characteristics of the enrolled studies

Study (Reference)	Year	Type of surgery /procedure	Patient age range & ASA status	Patients enrolled (Gender: F/M, n)	Nebulized Ketamine Group (dose, n)	Control Group (dose, n)	Scale used for sedation measurement	Outcomes
Nebulized Ketamine vs. Placebo Control								
Abdel-Ghaffar HS et al. [21]	2019	Elective tonsillectomy	7–12 years, ASA I-II	75 (40/35)	2 mg/kg, n = 25; 1 mg/kg, n = 25	saline 0.9% up to 3 ml, n = 25	5-point scales	I, VI
Nebulized Ketamine vs. Oral Ketamine								
Kamel AAF et al. [22]	2020	Elective surgery	3–6 years, ASA I-II	62 (24/38)	3 mg/kg, n = 31	10 mg/kg ketamine orally, n = 31	5-point scales	I, II, VI
Nebulized Ketamine vs. Intravenous Ketamine								
Abdel-Ghaffar HS et al. [21]	2019	Elective tonsillectomy	7–12 years, ASA I-II	75 (40/35)	2 mg/kg, n = 25; 1 mg/kg, n = 25	0.5 mg/kg ketamine i.v.,n = 25	5-point scales	I, VI
Nebulized Ketamine vs. Nebulized Midazolam								
Abdel-Ghaffar HS et al. [23]	2018	Bone marrow aspiration and biopsy	3–7 years, ASA I-II	60 (27/33)	2 mg/kg, n = 30	0.2 mg/kg nebulized midazolam, n = 30	5-point scales	I-III, VI
Verma I et al. [24]	2021	Elective cardiac surgery	1–12 years, ASA II-III	60 (23/37)	5 mg/kg, n = 30	0.2 mg/kg nebulized midazolam, n = 30	5-point scales	VI, VII
Shereef KM et al. [25]	2022	Elective surgery	3–7 years, ASA I-II	61 (Not mentioned)	2 mg/kg, n = 31	0.2 mg/kg nebulized midazolam, n = 30	5-point scales	VII
Nebulized Ketamine vs. Nebulized Dexmedetomidine								
Zanaty OM et al. [14]	2015	Dental procedures	3–6 years, ASA I-II	40 (19/21)	2 mg/kg, n = 20	2 ug/kg nebulized dexmedetomidine, n = 20	7-point scales	I-III, V-VII
Abdel-Ghaffar HS et al. [23]	2018	Bone marrow aspiration and biopsy	3–7 years, ASA I-II	60 (30/30)	2 mg/kg, n = 30	0.2 ug/kg nebulized dexmedetomidine, n = 30	5-point scales	I-III, VI
Mohammad Hazem I et al. [26]	2020	Elective tonsillectomy	3–6 years, ASA I-II	50 (Not mentioned)	3 mg/kg, n = 25	3 ug/kg nebulized dexmedetomidine, n = 25	6-point scales	I-III
Geetha K et al. [27]	2022	Diagnostic MRI	2–8 years, ASA I-II	71 (36/35)	2 mg/kg, n = 36	2 ug/kg nebulized dexmedetomidine, n = 35	4-point scales	I, IV, VI
Shereef KM et al. [25]	2022	Elective surgery	3–7 years, ASA I-II	62 (Not mentioned)	2 mg/kg, n = 31	2 ug/kg nebulized dexmedetomidine, n = 31	5-point scales	VI, VII
Singariya G et al. [28]	2022	Hernia repair surgery	2–8 years, ASA I-II	70 (15/55)	2 mg/kg, n = 35	2 ug/kg nebulized dexmedetomidine, n = 35	6-point scales	I-III, V-VII
Nebulized Ketamine vs. Nebulized Dexmedetomidine plus Nebulized Ketamine								
Zanaty OM et al. [14]	2015	Dental procedures	3–6 years, ASA I-II	40 (20/20)	2 mg/kg, n = 20	Nebulized dexmedetomidine/ketamine (1 ug/kg + 1 mg/kg), n = 20	7-point scales	I-III, V-VI
Dharamkhele SA et al. [29]	2020	Elective surgery	3–10 years, ASA I-II	47 (11/36)	2 mg/kg, n = 23	Nebulized dexmedetomidine/ketamine (1 ug/kg + 1 mg/kg), n = 24	7-point scales	I-III
Mohammad Hazem I et al. [26]	2020	Elective tonsillectomy	3–6 years, ASA I-II	50 (Not mentioned)	3 mg/kg, n = 25	Nebulized dexmedetomidine/ketamine (1.5 ug/kg + 1.5 mg/kg), n = 25	6-point scales	I-III

Note: I-Number of children with satisfactory sedation (defined as acceptable venipuncture, acceptable diagnostic procedures, acceptable operations, etc.); II-Number of children with satisfactory separation from parents; III-Number of children with satisfactory mask acceptance; IV-Onset of sedation; V-Recovery time; VI-Various adverse effects (Vomiting, emergence agitation, hypotension, etc.); VII-Hemodynamic status

### Risk of bias assessment

**Fig. 2:**
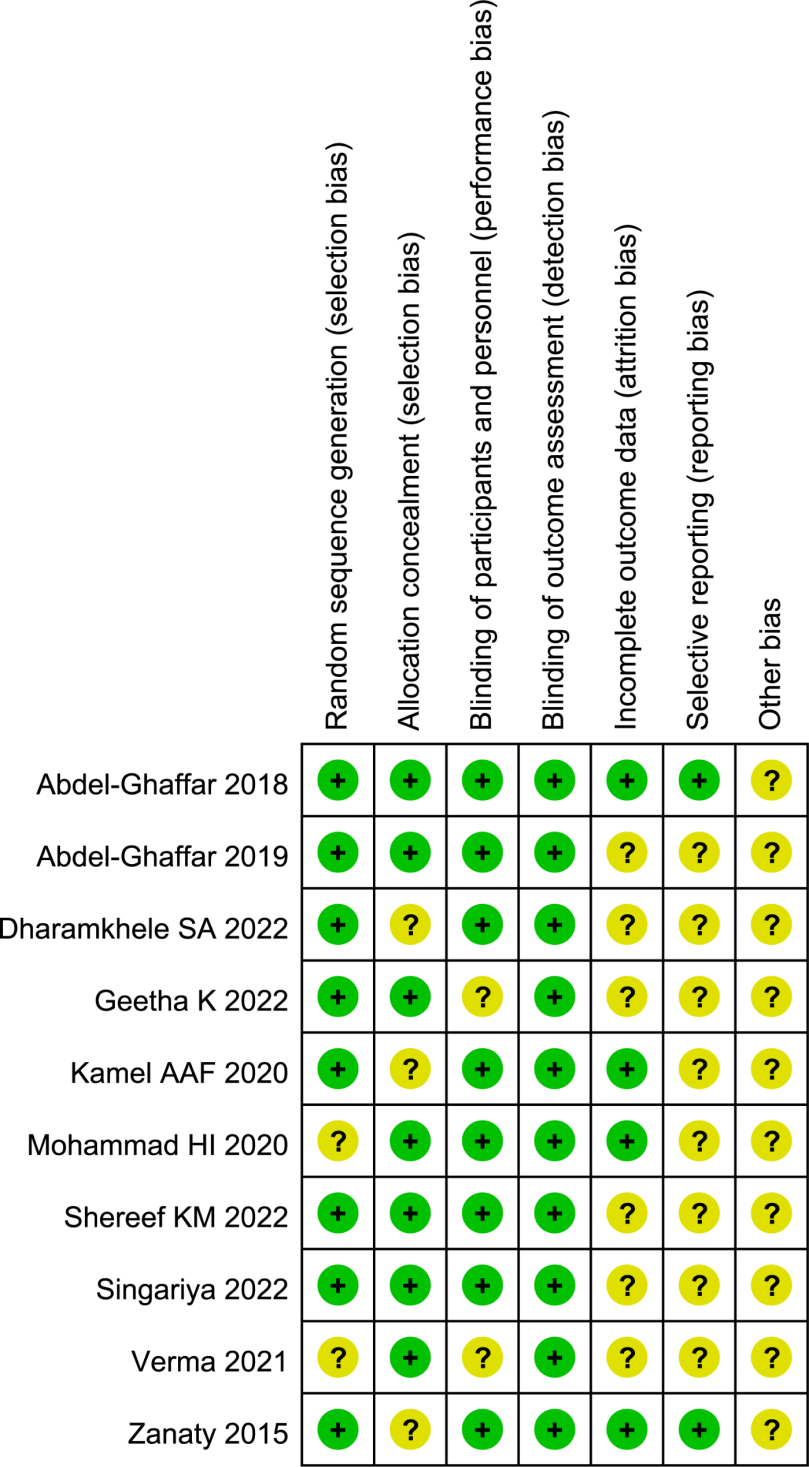
Risk of bias summary of included the trails: evaluation of bias risk items for each included study. Green circle, low risk of bias; red circle, high risk of bias; yellow circle, unclear risk of bias

Cochrane Collaboration’s risk of bias tool was employed to appraise the validity and quality of the RCTs by us. In all 10 enrolled studies, 8 studies (80.00%) delineated an appropriate method of random sequence generation, 7 studies (70.00%) reported adequate allocation concealment, 8 studies (80.00%) showed a low risk in blinding of participants and personnel domain, and all studies described the blinding procedure of outcome assessment. The detailed information about risk of bias assessment is presented in Fig. 2.

### Primary outcomes

#### Number of patients with satisfactory sedation levels

**Fig. 3:**
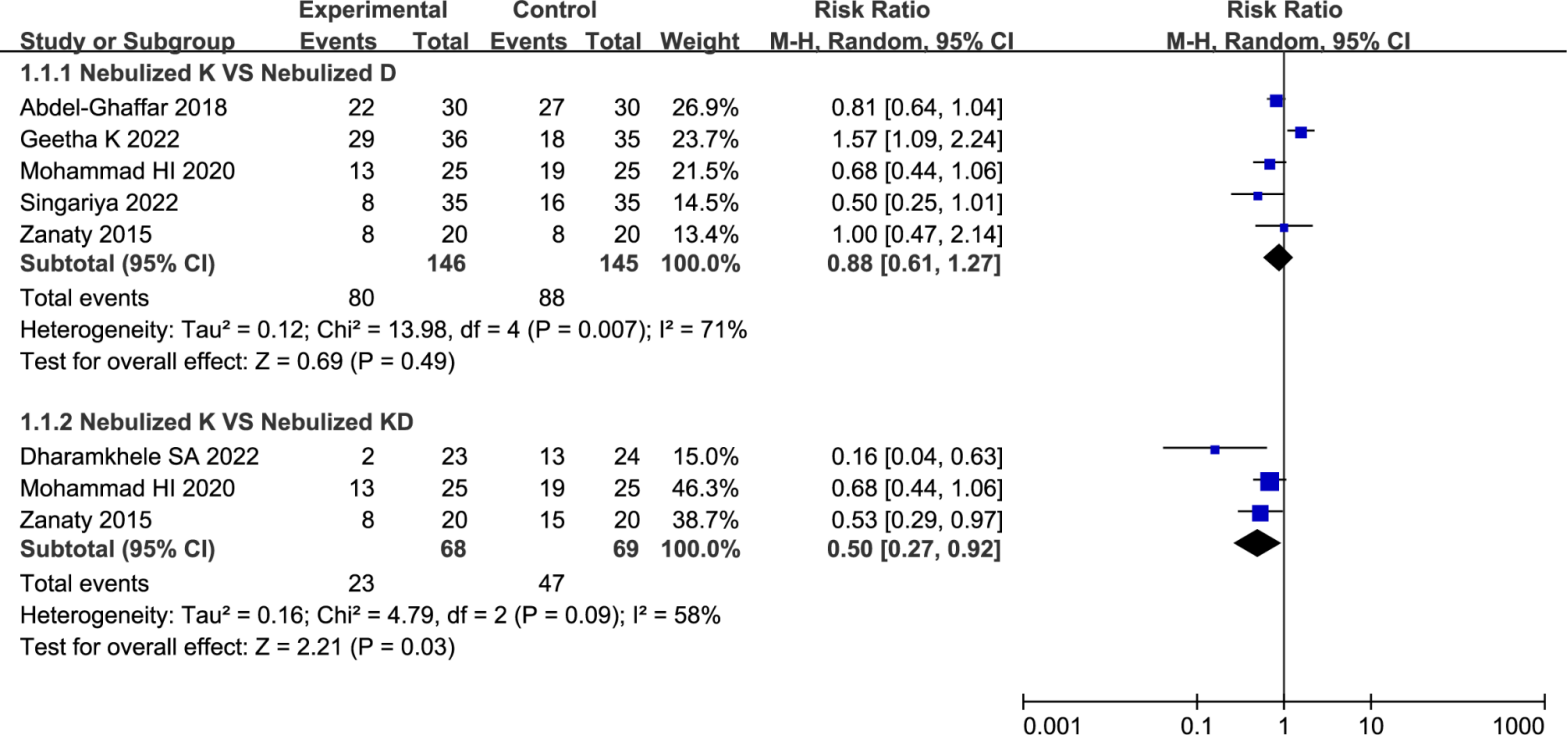
Forest plot: Number of children with satisfactory sedation. No significant differences were observed between nebulized ketamine group and nebulized dexmedetomidine group (RR = 0.88, with 95%CI [0.61, 1.27], *P* = 0.49); Nebulization of dexmedetomidine plus ketamine can provide better sedative effect than nebulized ketamine alone (RR = 0.50, with 95%CI [0.27, 0.92], *P* = 0.03)

Five studies compared nebulized ketamine to nebulized dexmedetomidine described the number of patients with satisfactory sedation levels [14, 23, 26–28]. Owing to existence of statistical heterogeneity, the random-effects model was chosen in present analysis. And the results indicated that no significant differences were observed between nebulized ketamine group and nebulized dexmedetomidine group (54.79% vs. 60.69%, RR = 0.88, with 95%CI [0.61, 1.27], *P* = 0.49, *I*^2^ = 71%; Fig. 3; Table 2). The sensitivity analysis indicated that the heterogeneity (*I*^2^ = 71%) derived from the Geetha K et al. study [27]. And heterogeneity was resolved (*I*^2^ = 0%) by omitting this study, the more reliable results indicated that the summary estimate was changed (46.36% vs. 63.64%, RR = 0.77, 95% CI [0.63, 0.94], *P* = 0.009).

**Table 2:** Outcomes

**Number of children with satisfactory sedation**						
Comparisons	Number of studies in analysis (Reference no.)	Patients in Nebulized K group (Incidence, %)	Patients in Control group (Incidence, %)	*I* ^2^	Risk ratio with [95% CI]	*P* Value
Nebulized K vs. Nebulized D	5 [14, 23, 26–28]	80/146 (54.79%)	88/145 (60.69%)	71%	0.88 [0.61, 1.27]	0.49
Nebulized K vs. Nebulized KD	3 [14, 26, 29]	23/68 (33.82%)	47/69 (68.11%)	58%	0.50 [0.27, 0.92]	**0.03**
Nebulized K vs. Nebulized M	1 [23]	22/30 (73.33%)	25/30 (83.33%)			
Nebulized K vs. Placebo Control	1 [21]	5/25 (20.00%)	0/25 (0.00%)			
Nebulized K vs. Oral K	1 [22]	9/31 (29.03%)	31/31 (100.00%)			
Nebulized K vs. Intravenous K	1 [21]	5/25 (20.00%)	0/25 (0.00%)			
**Number of children with satisfactory separation from parents**						
Comparisons	Number of studies in analysis (Reference no.)	Patients in Nebulized K group (Incidence, %)	Patients in Control group (Incidence, %)	*I* ^2^	Risk ratio with [95% CI]	*P* Value
Nebulized K vs. Nebulized D	4 [14, 23, 26, 28]	63/110 (57.27%)	81/110 (73.64%)	59%	0.81 [0.61, 1.08]	0.15
Nebulized K vs. Nebulized KD	3 [14, 26, 29]	44/68 (64.71%)	51/69 (73.91%)	57%	0.92 [0.74, 1.14]	0.42
Nebulized K vs. Nebulized M	1 [23]	21/30 (70.00%)	28/30 (93.33%)			
Nebulized K vs. Oral K	1 [22]	8/31 (25.81%)	31/31 (100.00%)			
**Number of children with satisfactory mask acceptance**						
Comparisons	Number of studies in analysis (Reference no.)	Patients in Nebulized K group (Incidence, %)	Patients in Control group (Incidence, %)	*I* ^2^	Risk ratio with [95% CI]	*P* Value
Nebulized K vs. Nebulized D	4 [14, 23, 26, 28]	41/110 (37.27%)	58/110 (52.73%)	50%	0.71 [0.45, 1.10]	0.13
Nebulized K vs. Nebulized KD	3 [14, 26, 29]	31/68 (45.59%)	49/69 (71.01%)	0%	0.69 [0.56, 0.86]	**0.001**
Nebulized K vs. Nebulized M	1 [23]	20/30 (66.67%)	17/30 (56.67%)			
**Onset of sedation**						
Comparisons	Number of studies in analysis (Reference no.)	Number of patients in Nebulized K group	Number of patients in in Control group	*I* ^2^	Mean difference with [95% CI]	*P* Value
Nebulized K vs. Nebulized D	1 [27]	36	35			
**Recovery time**						
Comparisons	Number of studies in analysis (Reference no.)	Number of patients in Nebulized K group	Number of patients in in Control group	*I* ^2^	Mean difference with [95% CI]	*P* Value
Nebulized K vs. Nebulized D	2 [14, 28]	55	55	98%	-2.96 [-8.69, 2.77]	0.31
**Vomiting**						
Comparisons	Number of studies in analysis (Reference no.)	Patients in Nebulized K group (Incidence, %)	Patients in Control group (Incidence, %)	*I* ^2^	Risk ratio with [95% CI]	*P* Value
Nebulized K vs. Nebulized D	3 [14, 23, 28]	6/85 (7.06%)	3/85 (3.53%)	0%	1.86 [0.53, 6.55]	0.34
Nebulized K vs. Nebulized KD	2 [14, 26]	2/45 (4.44%)	3/45 (6.67%)	31%	0.71 [0.15, 3.48]	0.68
Nebulized K vs. Nebulized M	2 [23, 24]	8/60 (13.33%)	1/60 (1.67%)	8%	5.67 [1.03, 31.20]	0.05
Nebulized K vs. Placebo Control	1 [21]	9/25 (36.00%)	2/25 (8.00%)			
Nebulized K vs. Intravenous K	1 [21]	9/25 (36.00%)	3/25 (12.00%)			
**Nystagmus**						
Comparisons	Number of studies in analysis (Reference no.)	Patients in Nebulized K group (Incidence, %)	Patients in Control group (Incidence, %)	*I* ^2^	Risk ratio with [95% CI]	*P* Value
Nebulized K vs. Oral K	1 [22]	1/31 (3.23%)	2/31 (6.45%)			
**Abnormal movement**						
Comparisons	Number of studies in analysis (Reference no.)	Patients in Nebulized K group (Incidence, %)	Patients in Control group (Incidence, %)	*I* ^2^	Risk ratio with [95% CI]	*P* Value
Nebulized K vs. Oral K	1 [22]	0/31 (0.00%)	1/31 (3.23%)			
**Hypersalivation**						
Comparisons	Number of studies in analysis (Reference no.)	Patients in Nebulized K group (Incidence, %)	Patients in Control group (Incidence, %)	*I* ^2^	Risk ratio with [95% CI]	*P* Value
Nebulized K vs. Nebulized D	2 [23, 28]	1/65 (1.54%)	1/65 (1.54%)	0%	1.00 [0.14, 6.94]	1.00
Nebulized K vs. Nebulized KD	1 [26]	0/25 (0.00%)	5/25 (20.00%)			
Nebulized K vs. Oral K	1 [22]	2/31 (0.00%)	3/31 (6.45%)			
Nebulized K vs. Nebulized M	1 [24]	9/30 (30.00%)	0/30 (0.00%)			
**Hypotension**						
Comparisons	Number of studies in analysis (Reference no.)	Patients in Nebulized K group (Incidence, %)	Patients in Control group (Incidence, %)	*I* ^2^	Risk ratio with [95% CI]	*P* Value
Nebulized K vs. Nebulized D	1 [14]	0/20 (0.00%)	2/20 (10.00%)			
Nebulized K vs. Nebulized KD	1 [14]	0/20 (0.00%)	0/20 (0.00%)			
**Bradycardia**						
Comparisons	Number of studies in analysis (Reference no.)	Patients in Nebulized K group (Incidence, %)	Patients in Control group (Incidence, %)	*I* ^2^	Risk ratio with [95% CI]	*P* Value
Nebulized K vs. Nebulized D	1 [14]	0/20 (0.00%)	2/20 (10.00%)			
Nebulized K vs. Nebulized KD	1 [14]	0/20 (0.00%)	0/20 (0.00%)			
**Emergence agitation**						
Comparisons	Number of studies in analysis (Reference no.)	Patients in Nebulized K group (Incidence, %)	Patients in Control group (Incidence, %)	*I* ^2^	Risk ratio with [95% CI]	*P* Value
Nebulized K vs. Nebulized D	4 [14, 23, 27, 28]	22/121 (18.18%)	4/120 (3.33%)	0%	4.98 [1.88, 13.16]	**0.001**
Nebulized K vs. Nebulized M	1 [23]	12/30 (26.67%)	6/30 (6.67%)			
Nebulized K vs. Nebulized DK	1 [14]	2/20 (10.00%)	1/20 (5.00%)			
**Hemodynamic parameters (Mean arterial pressure, MAP)**						
Comparisons	Number of studies in analysis (Reference no.)	Number of patients in Nebulized K group	Number of patients in in Control group	*I* ^2^	Mean difference with [95% CI]	*P* Value
Nebulized K vs. Nebulized M	2 [24, 25]	61	60	3%	3.35 [0.61, 6.09]	**0.02**
**Hemodynamic parameters (Heart rate, HR)**						
Comparisons	Number of studies in analysis (Reference no.)	Number of patients in Nebulized K group	Number of patients in in Control group	*I* ^2^	Mean difference with [95% CI]	*P* Value
Nebulized K vs. Nebulized D	1 [25]	31	31			

Three studies compared nebulized ketamine to nebulized ketamine plus dexmedetomidine reported the number of patients with satisfactory sedation levels [14, 26, 29]. Existence of statistical heterogeneity prompted us to applied random-effects model. The results indicated that nebulization of dexmedetomidine plus ketamine can provide better sedative effect than nebulized ketamine alone (33.82% vs. 68.11%, RR = 0.50, with 95%CI [0.27, 0.92], *P* = 0.03, *I*^2^ = 58%; Fig. 3; Table 2). The sensitivity analysis showed that the heterogeneity (*I*^2^ = 58%) was attributed to the Dharamkhele SA et al. [29] study. Following excluding this study, the heterogeneity was resolved (*I*^2^ = 0%), and the summary estimate was unchanged essentially (46.67% vs. 75.56%, RR = 0.63, 95% CI [0.44, 0.89], *P* = 0.009).

According to the GRADE summary of findings table, the quality of evidence pertaining to these outcomes was low. It was attributed to both inconsistency (*I*^*2*^ > 50%) and imprecision (lack of events number) (Table S1).

The results of Abdel-Ghaffar HS et al. [23] study indicated that no significant differences were observed between group midazolam and group ketamine (22/30 (73.33%) vs. 25/30 (83.33%); Table 2). According to Abdel-Ghaffar HS et al. [21] study, children in nebulized ketamine group showed more satisfactory sedation levels compared with children in the intravenous ketamine group (5/25 (20.00%) vs. 0/25 (0.00%); Table 2) and the control group (5/25 (20.00%) vs. 0/25 (0.00%); Table 2). However, the results of Kamel AAF et al. [22] study described that number of patients with satisfactory sedation levels was highly statistically significant difference in oral ketamine group than in nebulized ketamine group (9/31 (29.03%) vs. 31/31 (100.00%); Table 2).

#### Number of children with satisfactory separation from parents

**Fig. 4:**
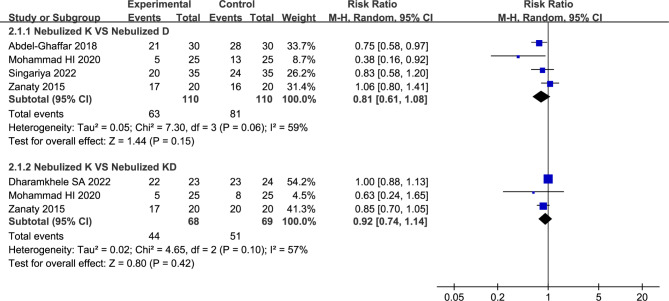
Forest plot: Number of children with satisfactory separation from parents. No significant differences were observed between nebulized ketamine group vs. nebulized dexmedetomidine group (RR = 0.81, with 95%CI [0.61, 1.08], *P* = 0.15), and nebulized ketamine group vs. nebulized ketamine plus dexmedetomidine group (RR = 0.92, with 95%CI [0.74, 1.14], *P* = 0.42)

Four studies compared nebulized ketamine to nebulized dexmedetomidine reported number of children with satisfactory separation from parents [14, 23, 26, 28]. The value of *I*^*2*^ (*I*^*2*^ = 59%) indicated that the statistical heterogeneity was existed, then we chose the random-effects model for analysis. Compared to nebulized dexmedetomidine, nebulized ketamine provided no obvious advantage in satisfactory separation from parents (57.27% vs. 73.64%, RR = 0.81, with 95%CI [0.61, 1.08], *P* = 0.15, *I*^*2*^ = 59%; Fig. 4; Table 2). After excluding the source of heterogeneity (Mohammad Hazem I et al. [26]), the heterogeneity was resolved (*I*^2^ = 39%) and the summary estimate was unchanged (68.24% vs. 80.00%, RR = 0.87, 95% CI [0.70, 1.08], *P* = 0.21).

Three studies compared nebulized ketamine to nebulized ketamine plus dexmedetomidine described the number of children with satisfactory separation from parents [14, 26, 29]. On account of existed statistical heterogeneity, the random-effects model was applied in present analysis. Analysis from the three studies found that nebulized ketamine plus dexmedetomidine has no statistical difference in number of children with satisfactory separation from parents compared to nebulized ketamine alone (64.71% vs. 73.91%, RR = 0.92, with 95%CI [0.74, 1.14], *P* = 0.42, *I*^*2*^ = 57%; Fig. 4; Table 2). Sensitivity analysis indicated that the heterogeneity (*I*^*2*^ = 57%) was attributable to the Dharamkhele SA et al. [29] study. Heterogeneity was resolved (*I*^2^ = 0%) by removing the study, and the summary estimate was unchanged (48.89% vs. 62.22%, RR = 0.84, with 95%CI [0.69, 1.03], *P* = 0.09, *I*^2^ = 0%).

The GRADE summary of findings table indicated that quality of evidence for present outcomes low. Inconsistency (*I*^*2*^ > 50%) and imprecision (limited number of events) were main factors (Table S1).

Abdel-Ghaffar HS et al. [23] found that no significant differences were observed between midazolam group and ketamine group(21/30 (70.00%) vs. 28/30 (93.33%); Table 2) in number of children with satisfactory separation from parents. Kamel AAF et al. [22] found that number of patients with satisfactory sedation levels was highly statistically significant difference in oral ketamine group than in nebulized ketamine group (8/31 (25.81) vs. 31/31 (100.00%); Table 2). Abdel-Ghaffar HS et al. [21] study indicated that patients in nebulized ketamine groups showed higher sedation scores compared with patients in the intravenous ketamine group (0.5 mg/kg) and the control group (*P* = 0.041), and there was no significant difference between nebulized ketamine group 1 (1 mg/kg) and nebulized ketamine group 2 (2 mg/kg) (*P* = 0.763).

#### Number of children with satisfactory mask acceptance

**Fig. 5:**
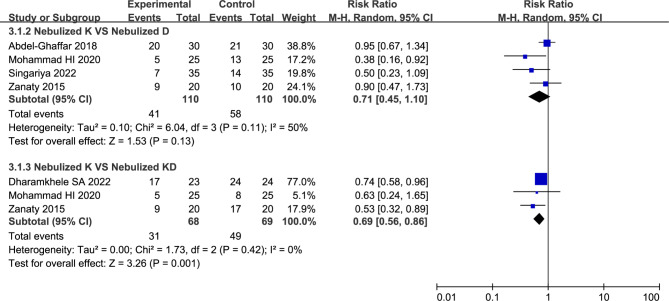
Forest plot: Number of children with satisfactory mask acceptance. No significant differences were observed between nebulized ketamine group and nebulized dexmedetomidine group (RR = 0.71, with 95%CI [0.45, 1.10], *P* = 0.13); Nebulized ketamine plus dexmedetomidine was associated with more satisfactory mask acceptance in pediatric patients compared to nebulized ketamine alone (RR = 0.69, with 95%CI [0.56, 0.86], *P* = 0.001)

Four studies compared nebulized ketamine to nebulized dexmedetomidine described number of children with satisfactory mask acceptance [14, 23, 26, 28]. We applied random-effects model in analysis as the existed statistical heterogeneity (*I*^2^ = 50%). Analysis from the four studies found that no significant differences were observed between Nebulized Ketamine Group and Nebulized Dexmedetomidine Group (37.27% vs. 52.73%, RR = 0.71, with 95%CI [0.45, 1.10], *P* = 0.13, *I*^*2*^ = 50%; Fig. 5; Table 2). Sensitivity analysis showed that the heterogeneity (*I*^*2*^ = 50%) was attributable to the Mohammad Hazem I et al. [26] study. After omitting this study, the heterogeneity was resolved (*I*^2^ = 20%) and the summary estimate was unchanged (42.35% vs. 52.94%, RR = 0.84, with 95%CI [0.59, 1.19], *P* = 0.32, *I*^2^ = 20%).

The GRADE summary of findings table showed that quality of evidence for this outcome was low. Inconsistency (*I*^2^ > 50%) and imprecision (lack of events number) were considered as main reasons (Table S1).

Three studies compared nebulized ketamine to nebulized ketamine plus dexmedetomidine described the number of children with satisfactory mask acceptance [14, 26, 29]. Given that no statistical heterogeneity (*I*^2^ = 0%) was detected, the fixed-effects model was used for analysis. The results indicated that using of ketamine plus dexmedetomidine via nebulization was associated with more satisfactory mask acceptance in pediatric patients compared to nebulized ketamine alone (45.59% vs. 71.01%, RR = 0.69, with 95%CI [0.56, 0.86], *P* = 0.001, *I*^2^ = 0%; Fig. 5; Table 2).

According to GRADE summary of findings table, quality of evidence for present outcome was moderate. The imprecision (lack of events number) was considered as the main reason (Table S1).

In addition, the results of Abdel-Ghaffar HS et al. study [23] indicated that no significant differences were observed between midazolam group and ketamine group (20/30 (66.67%) vs. 17/30 (56.67%); Table 2) in number of children with satisfactory mask acceptance.

### Secondary outcomes

Results of secondary outcomes including onset of sedation, recovery time, various adverse effects and hemodynamic status were summarized in Table 2.

#### Onset of sedation and recovery time

Geetha K et al. [27] found that the time to onset of sedation was significantly less in nebulized dexmedetomidine group compared to nebulized ketamine group (19.73 ± 8.43 min vs. 26.00 ± 7.33 min, *P* = 0.002). However, analysis of two studies found that no significant differences were observed between nebulized ketamine group and nebulized dexmedetomidine group in recovery time (MD = -2.96, with 95% CI [-8.69, 2.77], *P* = 0.31, *I*^2^ = 98%; Table 2).

#### Various adverse effects

**Fig. 6:**
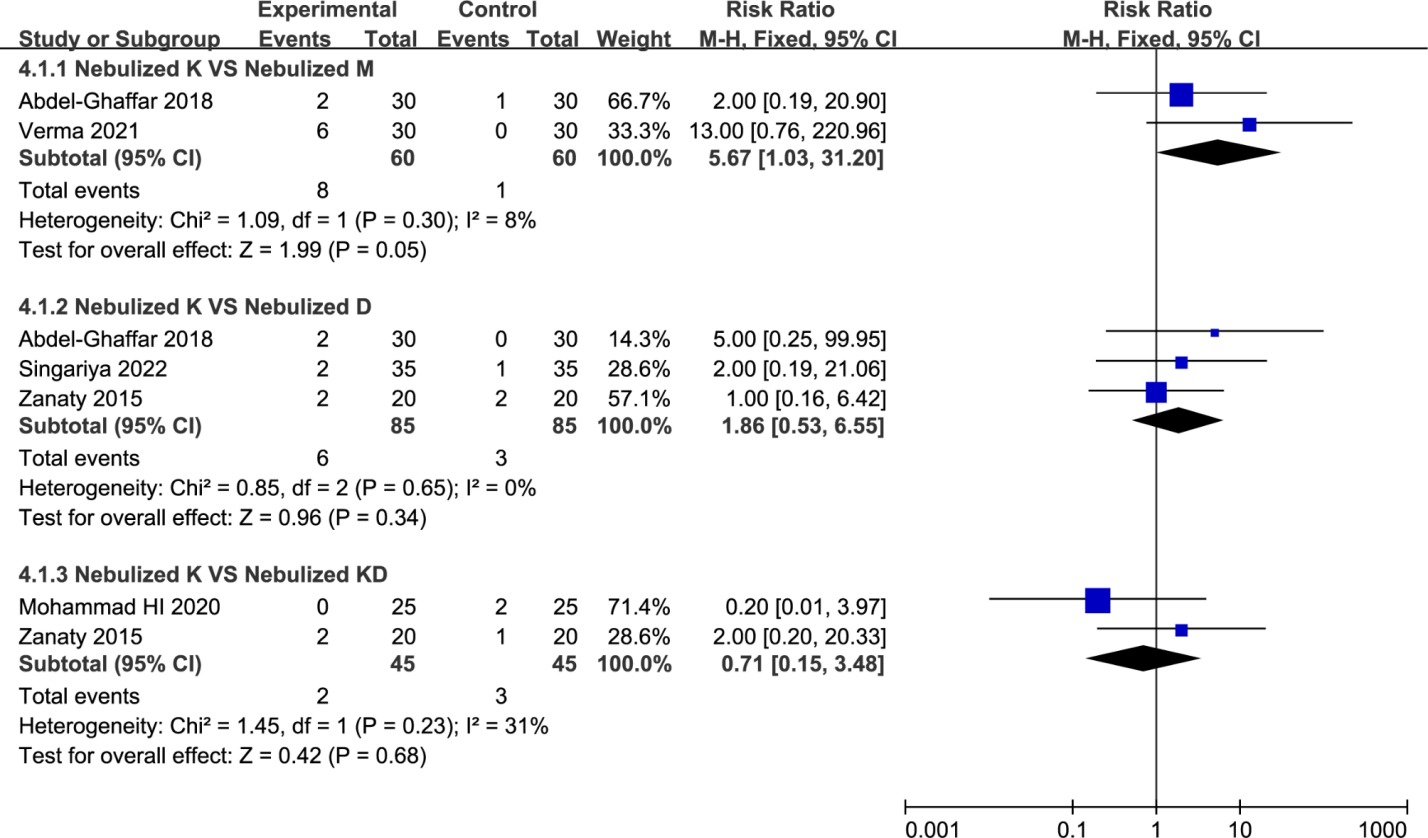
Forest plot: Incidence of Vomiting. No significant differences were observed between nebulized ketamine group vs. nebulized midazolam group (RR = 5.67, with 95%CI [1.03, 31.20], *P* = 0.05), nebulized ketamine group vs. nebulized dexmedetomidine group (RR = 1.86, with 95%CI [0.53, 6.55], *P* = 0.34), and nebulized ketamine group vs. nebulized ketamine plus dexmedetomidine group (RR = 0.71, with 95%CI [0.15, 3.48], *P* = 0.68)

The results involving various adverse effects indicated that no significant differences were found between nebulized ketamine group and nebulized dexmedetomidine group in incidence of vomiting (7.06% vs. 3.53%, RR = 1.86, with 95%CI [0.53, 6.55], *P* = 0.34,*I*^2^ = 0%; Fig. 6; Table 2), and nebulized ketamine was associated with higher incidence of emergence agitation (18.18% vs. 3.33%, RR = 4.98, with 95%CI [1.88, 13.16], *P* **=** 0.001, *I*^2^ = 0%; Table 2) compared to nebulized dexmedetomidine. And no significant differences were observed between nebulized ketamine group vs. nebulized midazolam group (13.33% vs. 1.67%, RR = 5.67, with 95%CI [1.03, 31.20], *P* = 0.05, *I*^2^ = 8%; Fig. 6) and nebulized ketamine group vs. nebulized ketamine plus dexmedetomidine group (4.44% vs. 6.67%, RR = 0.71, with 95%CI [0.15, 3.48], *P* = 0.68, *I*^2^ = 31%; Fig. 6) in the incidence of vomiting. In addition, for the occurrence of other adverse effects (e.g., hypotension, bradycardia, abnormal movement, nystagmus), the existing evidence was still lacking, and it was difficult to judge whether nebulized ketamine brings benefits compared with other sedative approaches.

#### Hemodynamic parameters

The results of general hemodynamic parameters indicated that nebulized ketamine provided more steady value of MAP (MD = 3.35, with 95% CI [0.61, 6.09], *P* = 0.02, *I*^2^ = 3%; Table 2) after administration compared to nebulized midazolam. And according to Shereef KM et al. study [25], the hemodynamic parameters (HR and MAP) showed statistically significant decrease throughout the perioperative period in nebulized dexmedetomidine group when compared with nebulized ketamine group.

## Discussion

As a recent technique, nebulized medication delivery provides improved usability issues and better bioavailability data [30] compared with common intranasal administration. In addition, Primosch et al. [31] suggested that administration by atomization is associated with significantly less adverse behaviors compared with administration by conventional drops in children undergoing dental procedures. Therefore, in order to estimate the effects of nebulized ketamine in pediatric sedation, the present study comparing the efficacy and safety of nebulized ketamine versus different pharmacological approaches was conducted by us.

Abdel-Ghaffar et al. [21] demonstrated that children who received nebulized ketamine achieved better sedation scores than those who received either placebo or intravenous ketamine. However, Kamel AAF et al. [22] found that oral ketamine as premedication is more effective than nebulized ketamine in producing more satisfactory sedation. The contradictory findings reported from the two aforementioned literatures were confusing. Investigation from Jonkman et al. [32] study on the bioavailability of inhaled ketamine may shed light on this issue. They found that the substantial reduction in bioavailability of nebulized ketamine could be attributed to residual liquid ketamine that remained in the nebulizer container, or aerosolized ketamine that adhered to the mouthpiece, or the large inhaled aerosol particles that trapped in the oropharynx. The available literatures regarding nebulized ketamine in comparison to other routes of administration remain limited. Its superiority over other routes such as oral and intravenous injection requires further investigation.

In addition, existing evidences indicated that nebulized ketamine provides inconspicuous advantages in sedative effects in children compared to nebulized dexmedetomidine. The results of co-primary outcomes in our study (including number of children with satisfactory sedation, number of children with satisfactory separation from parents, and number of children with satisfactory mask acceptance) showed that the differences among such two treatments were not significant. Our findings regarding adverse reactions indicated that nebulized dexmedetomidine may be a more appropriate option for pediatric sedation than nebulized ketamine due to its lower incidence of emergence agitation. Ketamine injection contains the preservative benzothonium chloride (BCl), which is often considered to be neurotoxic and is associated with a series of adverse reactions [33]. Meanwhile, Vranken JH et al. believed that preservative free ketamine might also be neurotoxic [34]. According to recent literatures, preservative-free s-ketamine has been applied in pediatric sedation or analgesia via intravenous, nasal drop, and rectal administration [35–37]. However, for included clinical trials in our present study, no researchers used preservative-free s-ketamine for nebulization in pediatric sedation. Therefore, whether preservative-free s-ketamine administrated via nebulization can reduce adverse reactions remains question for further study. Our study also found that administration of dexmedetomidine was associated with intense decrease in hemodynamic parameters (HR and MAP), which may be derived from the biphasic effects of α2-adrenoceptor [38]. And it was still accepted as a viable sedative option for pediatric patients in some studies, as such great hemodynamic changes could be mitigated by decelerating the rate of drug infusion [39, 40]. Moreover, the present study has demonstrated that the co-administration of dexmedetomidine and ketamine via nebulization can produce a more pronounced sedative effect compared to nebulization of ketamine alone. This finding indicates the potential significance of investigating the combined use of these two agents in future research endeavors.

One limitation in present study would be widespread low quality in the majority of outcomes assessed by the GRADE system, which might be mainly attributed to inconsistency (high heterogeneity) and imprecision (lack of events number). A systematic review of studies brings together material with an element of diversity. They differ in design and conduct as well as in participants, interventions, exposures, etc., and such diversity is commonly referred to as methodological or clinical heterogeneity [19]. Considering that high heterogeneity might add uncertainty to the results and influence the conclusions of the meta-analysis, subgroup or sensitivity analyses should be performed to determine the source of variation [41]. For substantial heterogeneity (present at *I*^2^ > 50%) existing in our present study, the sensitivity analysis was considered by us through omitting each study separately and we finally determined these origins of heterogeneity. In addition, although the thorough search strategy and an additional source from Google scholar were considered by us to ensure comprehensive coverage of the relevant literature, the number of enrolled pediatric patients was still insufficient in present study.

Therefore, it is imperative to conduct studies with large sample sizes in future to generate more dependable conclusions. Moreover, due to the fact that each outcome in the present study encompassed fewer than 10 studies, data for publication bias analysis were insufficient and we did not conduct it [20].

## Conclusions

Nebulized ketamine has been found to provide inconspicuous advantages in sedative effects to nebulized dexmedetomidine, and it is associated with a relatively high incidence of emergence agitation. Combination of nebulized ketamine and dexmedetomidine might be considered as one preferred option in pediatric sedation as it can provide more satisfactory sedative effects. However, the evidence available to date is insufficient to compare nebulized ketamine with ketamine administered through other routes or with other sedatives. The GRADE system indicated that overall quality of evidences was low or moderate, therefore, future studies with larger sample sizes and high quality are required to obtain more reliable conclusions.

### Electronic supplementary material

Below is the link to the electronic supplementary material.

Supplementary Material 1

Supplementary Material 2

## Data Availability

All data generated or analyzed during this study are included in this published article [and its supplementary information files].

## Abbreviations

RCTs: Randomized controlled trials
MD: Mean difference
CI: Confidence interval
RR: Risk ratio
NMDA: N-Methyl-D-Aspartate antagonist
MAP: Mean arterial pressure
HR: Heart rate
GRADE: Grading of recommendations assessment, development, and evaluation
PRISMA: Preferred reporting items for systematic reviews and meta-analyses statement
