# Supplementary material for: Comparison of the effects of ketamine via nebulization versus different pharmacological approaches in pediatric sedation: a systematic review and meta-analysis of randomized controlled trials
Source: BMC Anesthesiol. 2023 Nov 16;23:375. doi: 10.1186/s12871-023-02298-4 (PMC10652489; doi:10.1186/s12871-023-02298-4)
Supplement: Supplementary file 1 — Supplementary Material 1 [file 12871_2023_2298_MOESM1_ESM.docx]

**Table S1. GRADE summary of findings table**

| **Quality assessment** | | | | | | | **Summary of Findings** | | | | |
| --- | --- | --- | --- | --- | --- | --- | --- | --- | --- | --- | --- |
| **Participants (studies) Follow up** | **Risk of bias** | **Inconsistency** | **Indirectness** | **Imprecision** | **Publication bias** | **Overall quality of evidence** | **Study event rates (%)** | | **Relative effect** (95% CI) | **Anticipated absolute effects** | |
|  |  |  |  |  |  |  | **With Control** | **With Number of children with satisfactory sedation** |  | **Risk with Control** | **Risk difference with Number of children with satisfactory sedation** (95% CI) |
| **Number of children with satisfactory sedation**  **Nebulized K VS Control - Nebulized K VS Nebulized D** (CRITICAL OUTCOME) | | | | | | | | | | | |
| 291 (5 studies) | no serious risk of bias | serious^1^ | no serious indirectness | serious^2^ | undetected | ⊕⊕⊝⊝ **LOW**^1,2^ due to inconsistency, imprecision | 88/145  (60.7%) | 80/146  (54.8%) | **RR 0.88**  (0.61 to 1.27) | **Study population** | |
|  |  |  |  |  |  |  |  |  |  | **607 per 1000** | **73 fewer per 1000** (from 237 fewer to 164 more) |
|  |  |  |  |  |  |  |  |  |  | **Moderate** | |
|  |  |  |  |  |  |  |  |  |  | **514 per 1000** | **62 fewer per 1000** (from 200 fewer to 139 more) |
| **Number of children with satisfactory sedation**  **Nebulized K VS Control - Nebulized K VS Nebulized KD** (CRITICAL OUTCOME) | | | | | | | | | | | |
| 137 (3 studies) | no serious risk of bias | serious^1^ | no serious indirectness | serious^2^ | undetected | ⊕⊕⊝⊝ **LOW**^1,2^ due to inconsistency, imprecision | 47/69  (68.1%) | 23/68  (33.8%) | **RR 0.5**  (0.27 to 0.92) | **Study population** | |
|  |  |  |  |  |  |  |  |  |  | **681 per 1000** | **341 fewer per 1000** (from 54 fewer to 497 fewer) |
|  |  |  |  |  |  |  |  |  |  | **Moderate** | |
|  |  |  |  |  |  |  |  |  |  | **750 per 1000** | **375 fewer per 1000** (from 60 fewer to 548 fewer) |
| **Satisfactory separation from parents**  **Nebulized K VS Control - Nebulized K VS Nebulized D** (CRITICAL OUTCOME) | | | | | | | | | | | |
| 220 (4 studies) | no serious risk of bias | serious^1^ | no serious indirectness | serious^2^ | undetected | ⊕⊕⊝⊝ **LOW**^1,2^ due to inconsistency, imprecision | 81/110  (73.6%) | 63/110  (57.3%) | **RR 0.81**  (0.61 to 1.08) | **Study population** | |
|  |  |  |  |  |  |  |  |  |  | **736 per 1000** | **140 fewer per 1000** (from 287 fewer to 59 more) |
|  |  |  |  |  |  |  |  |  |  | **Moderate** | |
|  |  |  |  |  |  |  |  |  |  | **743 per 1000** | **141 fewer per 1000** (from 290 fewer to 59 more) |
| **Satisfactory separation from parents**  **Nebulized K VS Control - Nebulized K VS Nebulized KD** (CRITICAL OUTCOME) | | | | | | | | | | | |
| 137 (3 studies) | no serious risk of bias | serious^1^ | no serious indirectness | serious^2^ | undetected | ⊕⊕⊝⊝ **LOW**^1,2^ due to inconsistency, imprecision | 51/69  (73.9%) | 44/68  (64.7%) | **RR 0.92**  (0.74 to 1.14) | **Study population** | |
|  |  |  |  |  |  |  |  |  |  | **739 per 1000** | **59 fewer per 1000** (from 192 fewer to 103 more) |
|  |  |  |  |  |  |  |  |  |  | **Moderate** | |
|  |  |  |  |  |  |  |  |  |  | **958 per 1000** | **77 fewer per 1000** (from 249 fewer to 134 more) |
| **Satisfactory mask acceptance**  **Nebulized K VS Control - Nebulized K VS Nebulized D** (CRITICAL OUTCOME) | | | | | | | | | | | |
| 220 (4 studies) | no serious risk of bias | serious^1^ | no serious indirectness | serious^2^ | undetected | ⊕⊕⊝⊝ **LOW**^1,2^ due to inconsistency, imprecision | 58/110  (52.7%) | 41/110  (37.3%) | **RR 0.71**  (0.45 to 1.1) | **Study population** | |
|  |  |  |  |  |  |  |  |  |  | **527 per 1000** | **153 fewer per 1000** (from 290 fewer to 53 more) |
|  |  |  |  |  |  |  |  |  |  | **Moderate** | |
|  |  |  |  |  |  |  |  |  |  | **510 per 1000** | **148 fewer per 1000** (from 280 fewer to 51 more) |
| **Satisfactory mask acceptance**  **Nebulized K VS Control - Nebulized K VS Nebulized KD** (CRITICAL OUTCOME) | | | | | | | | | | | |
| 137 (3 studies) | no serious risk of bias | no serious inconsistency | no serious indirectness | serious^2^ | undetected | ⊕⊕⊕⊝ **MODERATE**^2^ due to imprecision | 49/69  (71%) | 31/68  (45.6%) | **RR 0.69**  (0.56 to 0.86) | **Study population** | |
|  |  |  |  |  |  |  |  |  |  | **710 per 1000** | **220 fewer per 1000** (from 99 fewer to 312 fewer) |
|  |  |  |  |  |  |  |  |  |  | **Moderate** | |
|  |  |  |  |  |  |  |  |  |  | **850 per 1000** | **264 fewer per 1000** (from 119 fewer to 374 fewer) |

^1^ *I*^2^ > 50%
^2^ Total number of events is less than 300
