# Supplementary material for: Comparison of the effects of ketamine via nebulization versus different pharmacological approaches in pediatric sedation: a systematic review and meta-analysis of randomized controlled trials
Source: BMC Anesthesiol. 2023 Nov 16;23:375. doi: 10.1186/s12871-023-02298-4 (PMC10652489; doi:10.1186/s12871-023-02298-4)
Supplement: Supplementary file 2 — Supplementary Material 2 [file 12871_2023_2298_MOESM2_ESM.docx]

**Appendix S1 Search Strategy**

Electronic databases including PubMed, Embase, and Cochrane Library were searched by us. The last literature search was performed on Feb 27, 2023. The details of search strategy are as follows:

**Pubmed**

#1 ((((((Infant [mesh] OR (newborn* [tiab] or neonat* [tiab] or infant* [tiab] or infancy [tiab] or baby [tiab] or babies [tiab] or toddler* [tiab])) OR (Child [mesh] OR Pediatrics [mesh] OR (p?ediatric* [tiab] or child* [tiab] or kindergar* [tiab] or preschool* [tiab] or kid [tiab] or kids [tiab] or schoolchild* [tiab] or "school age" [tiab] or schoolage [tiab] or preteen* [tiab] or youth* [tiab] or prepubescent* [tiab]))) OR (Adolescent [mesh] OR (adolesc* [tiab] or teen* [tiab] or youth* [tiab] or underage* [tiab] or "under age*" [tiab] or minor* [tiab] or juvenile* [tiab] or pubert* [tiab] or pubescen* [tiab] or "young people*" [tiab] or "young person*" [tiab] or "young adult*" [tiab])))

#2 inhal* [tiab]

#3 aerosoli* [tiab]

#4 nebuli* [tiab]

#5 atomi* [tiab]

#6 #2 OR #3 OR #4 OR #5

#7 ketamine [MeSH] OR ketamine [tiab]

#8 #1 AND #6 AND #7

**Embase**

#1 'infant'/exp

#2 newborn*:ab,ti OR neonat*:ab,ti OR infant*:ab,ti OR infancy:ab,ti OR baby:ab,ti OR babies:ab,ti OR toddler*:ab,ti

#3 #1 OR #2

#4 'child'/exp

#5 'pediatrics'/exp

#6 paediatric*or:ab,ti AND pediatric*:ab,ti OR child*:ab,ti OR kindergar*:ab,ti OR preschool*:ab,ti OR kid:ab,ti OR kids:ab,ti OR schoolchild*:ab,ti OR 'school age':ab,ti OR schoolage:ab,ti OR preteen*:ab,ti OR youth*:ab,ti OR prepubescent*:ab,ti

#7 #4 OR #5 OR #6

#8 'adolescent'/exp

#9 adolesc*:ab,ti OR teen*:ab,ti OR youth*:ab,ti OR underage*:ab,ti OR 'under age*':ab,ti OR minor*:ab,ti OR juvenile*:ab,ti OR pubert*:ab,ti OR pubescen*:ab,ti OR 'young people*':ab,ti OR 'young person*':ab,ti OR 'young adult*':ab,ti

#10 #8 OR #9

#11 (#3 OR #7 OR #10) AND [embase]/lim

#12 'randomized controlled trial'/exp

#13 'controlled clinical trial'/exp

#14 'randomization'/exp

#15 'double blind procedure'/exp

#16 'single blind procedure'/exp

#17 #12 OR #13 OR #14 OR #15 OR #16

#18 'human'/exp

#19 #17 AND #18 AND [embase]/lim

#20 'ketamine'/exp

#21 'ketamine':ab,ti

#22 (#20 OR #21) AND [embase]/lim

#23 (inhal* OR aerosoli* OR nebuli* OR atomi*):ab,ti

#24 #23 AND [embase]/lim

#25 #11 AND #19 AND #22 AND #24

**Cochrane**

#1 MeSH descriptor: [Infant] explode all trees

#2 (newborn* or neonat* or infant* or infancy or baby or babies or toddler*):ti,ab,kw

#3 #1 or #2

#4 MeSH descriptor: [Child] explode all trees

#5 MeSH descriptor: [Pediatrics] explode all trees

#6 (paediatric*or pediatric* or child* or kindergar* or preschool* or kid or kids or schoolchild* or 'school age' or schoolage or preteen* or youth* or prepubescent*):ti,ab,kw

#7 #4 or #5 or #6

#8 MeSH descriptor: [Adolescent] explode all trees

#9 (adolesc* or teen* or youth* or underage* or "under age*" or minor* or juvenile* or pubert* or pubescen* or "young people*" or "young person*" or "young adult*"):ti,ab,kw

#10 #8 or #9

#11 #3 or #7 or #10

#12 MeSH descriptor: [ketamine] explode all trees

#13 (ketamine*):ti,ab,kw

#14 #12 or #13

#15 (inhal* or aerosoli* or nebuli* or atomi*):ti,ab,kw

#16 #11 and #14 and #15
